# Clinical characteristics, risk factors and outcomes of cancer patients with COVID‐19: A population‐based study

**DOI:** 10.1002/cam4.4888

**Published:** 2022-05-31

**Authors:** Jiandong Zhou, Ishan Lakhani, Oscar Chou, Keith Sai Kit Leung, Teddy Tai Loy Lee, Michelle Vangi Wong, Zhen Li, Abraham Ka Chung Wai, Carlin Chang, Ian Chi Kei Wong, Qingpeng Zhang, Gary Tse, Bernard Man Yung Cheung

**Affiliations:** ^1^ Nuffield Department of Medicine University of Oxford Oxford UK; ^2^ Cardiovascular Analytics Group, Laboratory of Cardiovascular Physiology Hong Kong China; ^3^ Li Ka Shing Faculty of Medicine University of Hong Kong Hong Kong China; ^4^ Aston Medical School Aston University Birmingham UK; ^5^ Emergency Medicine Unit University of Hong Kong Hong Kong China; ^6^ Department of Radiology Tongji Hospital of Tongji Medical College of Huazhong University of Science and Technology Wuhan China; ^7^ Division of Neurology, Department of Medicine University of Hong Kong Hong Kong China; ^8^ Department of Pharmacology and Pharmacy University of Hong Kong Hong Kong China; ^9^ Medicines Optimisation Research and Education (CMORE) UCL School of Pharmacy London UK; ^10^ Tianjin Key Laboratory of Ionic‐Molecular Function of Cardiovascular Disease, Department of Cardiology, Tianjin Institute of Cardiology Second Hospital of Tianjin Medical University Tianjin China; ^11^ Kent and Medway Medical School Canterbury Kent UK; ^12^ Division of Clinical Pharmacology, Department of Medicine University of Hong Kong Hong Kong China

**Keywords:** cancer, COVID‐19, intensive care unit, intubation, mortality

## Abstract

**Introduction:**

Cancer patients may be susceptible to poorer outcomes in COVID‐19 infection owing to the immunosuppressant effect of chemotherapy/radiotherapy and cancer growth, along with the potential for nosocomial transmission due to frequent hospital admissions.

**Methods:**

This was a population‐based retrospective cohort study of COVID‐19 patients who presented to Hong Kong public hospitals between 1 January 2020 and 8 December 2020. The primary outcome was a composite endpoint of requirement for intubation, ICU admission and 30‐day mortality.

**Results:**

The following study consisted of 6089 COVID‐19 patients (median age 45.9 [27.8.1–62.7] years; 50% male), of which 142 were cancer subjects. COVID‐19 cancer patients were older at baseline and tended to present with a higher frequency of comorbidities, including diabetes mellitus, hypertension, chronic obstructive pulmonary disease, ischemic heart disease, ventricular tachycardia/fibrillation and gastrointestinal bleeding (*p* < 0.05). These subjects also likewise tended to present with higher serum levels of inflammatory markers, including D‐dimer, lactate dehydrogenase, high sensitivity troponin‐I and C‐reactive protein. Multivariate Cox regression showed that any type of cancer presented with an almost four‐fold increased risk of the primary outcome (HR: 3.77; 95% CI: 1.63–8.72; *p* < 0.002) after adjusting for significant demographics, Charlson comorbidity index, number of comorbidities, past comorbidities and medication history. This association remained significant when assessing those with colorectal (HR: 5.07; 95% CI: 1.50–17.17; *p* < 0.009) and gastrointestinal malignancies (HR: 3.79; 95% CI: 1.12–12.88; *p* < 0.03), but not with lung, genitourinary, or breast malignancies, relative to their respective cancer‐free COVID‐19 counterparts.

**Conclusions:**

COVID‐19 cancer patients are associated with a significantly higher risk of intubation, ICU admission and/or mortality.

## Graphical abstract

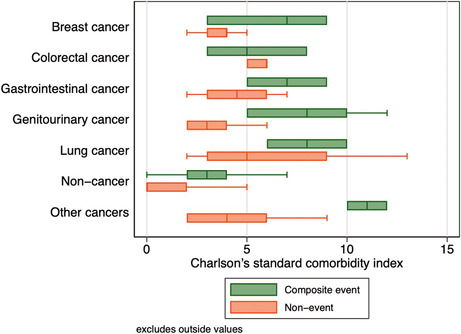

## 1 INTRODUCTION

The severe acute respiratory syndrome‐coronavirus‐2 (SARS‐CoV‐2) has spread rampantly worldwide, leading to the coronavirus disease 2019 (COVID‐19) pandemic that has burdened most healthcare systems. As of 14 June 2021, it is estimated that there are 175,306,598 global cases with 3,792,777 confirmed deaths.^1^ Since its inception, several studies have been conducted to assess the underlying pathogenesis of the disease as well as strategies for risk stratification, prognostic assessment and treatment. Although the majority of COVID‐19 patients only present with mild‐to‐moderate symptoms, namely fever, cough and fatigue, some cases do eventually develop more severe complications such as arrhythmias, respiratory failure, renal failure and shock.^2^

Regarding the different subgroups, the current school of thought follows that patients with underlying diseases are not only more susceptible to COVID‐19^3^ but also tend to present with worse long‐term outcomes following infection.^4^ As a result, cancer patients are particularly vulnerable to COVID‐19, primarily due to the immunosuppressive effect of cancer growth and antitumor medication, along with the enhanced risk for nosocomial transmission secondary to the repetitive hospital admissions or prolonged hospitalization of these subjects.^5^, ^6^, ^7^, ^8^ As such, there is an abundance of literature recently dedicated to understanding the implication of COVID‐19 in cancer patients, with specific emphasis on the risk of long‐term complications and subsequent clinical management. The following study aims to provide further insight on this topic by examining the prospective long‐term outcomes among COVID‐19 patients with different types of cancers from Hong Kong, China.

## 2 METHODS

### 2.1 Study population and their baseline characteristics

This population‐based retrospective cohort analysis is part of a larger study of antihypertensive drugs and infection outcomes that has been approved by the Institutional Review Board of the University of Hong Kong/Hospital Authority Hong Kong West Cluster. COVID‐19 patients who presented to Hong Kong public hospitals and outpatient clinics between 1 January 2020 and 8 December 2020 were identified using the territory‐wide Clinical Data Analysis and Reporting System (CDARS), which provides a central medium through which clinical data can be obtained for analysis. CDARS is a territory‐wide database that centralizes patient information from 43 local hospitals and their associated ambulatory and outpatient facilities to establish comprehensive medical data including clinical characteristics, disease diagnosis, laboratory results and drug treatment details. Our team has previously implemented this platform in various cohort studies,^9^ including those on COVID‐19.^10^, ^11^, ^12^, ^13^ In this context, CDARS was used to recruit patients who presented with positive real‐time polymerase chain reaction (RT‐PCR) for COVID‐19 conducted in Accident and Emergency as well as out‐ and in‐patient settings. This was followed by the retrieval of patient demographics, along with comprehensive medical records, namely details pertaining to disease diagnoses, prior comorbidities, medication treatments and several laboratory parameters, including complete blood counts, renal function tests, liver function tests, clotting profile, arterial blood gas, blood glucose and HbA1c, as well as various inflammatory markers. There was no adjudication of the outcomes as this relied on the ICD‐9 coding or a record in the death registry. However, the coding was performed by the clinicians or administrative staff, who were not involved in the mode development.

The inclusion criteria for this study were as follows: patients who had a positive RT‐PCR for COVID‐19, had preexisting active malignancy diagnosed by clinical, radiological and pathological investigations and were admitted to the public hospital within the stated time period. The exclusion criteria included malignancy diagnosed after COVID‐19 infection and patients in complete remission from cancer. Active cancer diagnosis was determined according to the ICD‐9 codes of cancers as presented in Table S1. The primary outcome was a composite endpoint of patients who required intubation, required intensive care unit (ICU) admission and/or suffered 30‐day mortality. Details regarding the individual components that constitute this composite endpoint were extracted from CDARS.

### 2.2 Statistical analysis

Descriptive statistics were reported either as median (interquartile range) or as count (percentage). The cohort of COVID‐19 patients was stratified according to the presence or the absence of cancer at baseline. Chi‐squared test with Yates' correction was used for 2 × 2 contingency data, with Pearson's Chi‐squared test instead employed for data with more than two categories. Differences between continuous variables were assessed using the Mann–Whitney *U* test. The association between various patient clinical and laboratory parameters with the composite outcome was examined using Cox proportional hazards model. Three stepwise multivariate models were constructed using variables that were significant in univariate analysis. This included an adjustment for significant demographics first, followed by the addition of Charlson's comorbidity index score, number of comorbidities and past comorbidities, and subsequently, the addition of medication treatments. All statistical tests were two‐tailed and considered significant if *p* value is <0.05. There was no imputation performed for missing data. No blinding was performed for the predictor as the values were obtained from the electronic health records automatically. All the statistical analysis and visualizations were performed using Stata (Version 13.0) RStudio software (Version: 1.1.456) and Python (Version: 3.6).

## 3 RESULTS

### 3.1 Baseline characteristics

The following study consisted of 6089 COVID‐19 patients (median age: 45.9 [27.8.1–62.7] years; 50% male), of which 142 were cancer subjects (Table 1). COVID‐19 cancer patients were older at baseline and tended to present with a higher Charlson's standard comorbidity index and overall frequency of comorbidities, including diabetes mellitus, hypertension, chronic obstructive pulmonary disease, stroke/transient ischemic attack, ischemic heart disease, ventricular tachycardia/fibrillation, dementia/Alzheimer's disease and gastrointestinal bleeding (*p* < 0.05). With regards to the complete blood count with differentials, COVID‐19 patients with cancer also had lower red blood cell, platelet and lymphocyte count (*p* < 0.05) compared with non‐cancer COVID‐19 patients. Furthermore, there were significantly greater differences in both liver and renal function in cancer subjects as cancer subjects had lower albumin levels, higher urea and protein levels (*p* < 0.05).

**TABLE 1:** Demographics and clinical characteristics of hospitalized COVID‐19 patients with/without cancer

Characteristics	Cancer (*N* = 142) Median (IQR) or Count (%)	No cancer (*N* = 5947) Median (IQR) or Count (%)	*p* value
Demographics			
Male gender	64 (45.07)	2966 (49.87)	0.5547
Baseline age, years	63.82 (51.01–78.62)	45.22 (27.62–62.33)	<0.0001
Past comorbidities			
Charlson's standard comorbidity index	4.0 (3.0–6.0)	0.0 (0.0–2.0)	<0.0001
Number of comorbidities	1.0 (1.0–2.0)	0.0 (0.0–0.0)	<0.0001
Diabetes mellitus	11 (7.74)	132 (2.21)	0.0001
Systemic embolism	1 (0.70)	16 (0.26)	0.87
Hypertension	54 (38.02)	845 (14.20)	<0.0001
Heart failure	1 (0.70)	31 (0.52)	0.7714
Atrial fibrillation	5 (3.52)	84 (1.41)	0.0951
Chronic renal failure	1 (0.70)	15 (0.25)	0.8358
Liver diseases	3 (2.11)	29 (0.48)	0.0423
Ventricular tachycardia/fibrillation	3 (2.11)	28 (0.47)	0.0366
Dementia and Alzheimer	3 (2.11)	22 (0.36)	0.0120
AMI	6 (4.22)	68 (1.14)	0.0045
COPD	6 (4.22)	75 (1.26)	0.0093
IHD	16 (11.26)	180 (3.02)	<0.0001
PVD	2 (1.40)	25 (0.42)	0.2716
Stroke/TIA	9 (6.33)	112 (1.88)	0.0009
Gastrointestinal bleeding	12 (8.45)	103 (1.73)	<0.0001
Obesity	1 (0.70)	23 (0.38)	0.9338
Medications			
ACEI	6 (4.22)	198 (3.32)	0.7415
ARB	11 (7.74)	179 (3.00)	0.0050
Calcium channel blockers	31 (21.83)	562 (9.45)	<0.0001
Beta blockers	17 (11.97)	245 (4.11)	<0.0001
Diuretics for hypertension	3 (2.11)	61 (1.02)	0.4113
Diuretics for heart failure	12 (8.45)	134 (2.25)	<0.0001
Nitrates	4 (2.81)	86 (1.44)	0.337
Antihypertensive drugs	8 (5.63)	101 (1.69)	0.0022
Antidiabetic drugs	19 (13.38)	274 (4.60)	<0.0001
Lipid‐lowering drugs	18 (12.67)	465 (7.81)	0.0783
Steroid	20 (14.08)	545 (9.16)	0.1012
Lopinavir/ritonavir	16 (11.26)	812 (13.65)	0.5496
Ribavirin	17 (11.97)	624 (10.49)	0.7108
Interferon beta‐1B	29 (20.42)	847 (14.24)	0.1025
Proton pump inhibitors	53 (37.32)	768 (12.91)	<0.0001
Famotidine	37 (26.05)	811 (13.63)	0.0007
Anticoagulants	31 (21.83)	468 (7.86)	<0.0001
Antiplatelets	20 (14.08)	381 (6.40)	0.0017
Complete blood counts			
Mean corpuscular volume, fL	88.2 (83.7–91.95)	87.2 (83.5–90.4)	0.0508
Basophil, ×10^9/L	0.01 (0.0–0.02)	0.01 (0.0–0.02)	0.0905
Eosinophil, ×10^9/L	0.01 (0.0–0.1)	0.03 (0.0–0.1)	0.2024
Lymphocyte, ×10^9/L	1.1 (0.76–1.5)	1.33 (0.97–1.8)	<0.0001
Blast, ×10^9/L	0.0 (0.0–0.0)	0.0 (0.0–0.0)	0.7039
Metamyelocyte, ×10^9/L	0.23 (0.12–0.27)	0.1 (0.07–0.17)	0.6589
Monocyte, ×10^9/L	0.5 (0.36–0.67)	0.5 (0.37–0.63)	0.8371
Neutrophil, ×10^9/L	3.24 (2.42–4.64)	3.2 (2.37–4.34)	0.4918
White cell count, ×10^9/L	5.2 (3.99–6.44)	5.34 (4.24–6.71)	0.2745
Mean cell hemoglobin, pg	30.4 (29.0–32.2)	29.9 (28.5–31.18)	0.0032
Myelocyte, ×10^9/L	0.04 (0.03–0.12)	0.22 (0.09–0.37)	0.0122
Platelet, ×10^9/L	197.0 (157.5–258.0)	215.0 (174.0–267.85)	0.0371
Reticulocyte, ×10^9/L	71.49 (50.39–86.84)	42.6 (30.2–71.5)	0.3115
Red blood count, ×10^12/L	4.23 (3.84–4.65)	4.65 (4.32–5.05)	<0.0001
Hematocrit, L/L	0.36 (0.33–0.39)	0.4 (0.37–0.43)	<0.0001

Abbreviations: ACEI, angiotensinogen‐converting enzyme inhibitor; AMI, acute myocardial infarction; APTT, activated partial thromboplastin time; ARB, angiotensin receptor blocker; COPD, chronic obstructive pulmonary disease; IHD, ischemic heart disease; PVD, peripheral vascular disease; TIA, transient ischemic attack.

### 3.2 Outcome analysis

**FIGURE 1:**
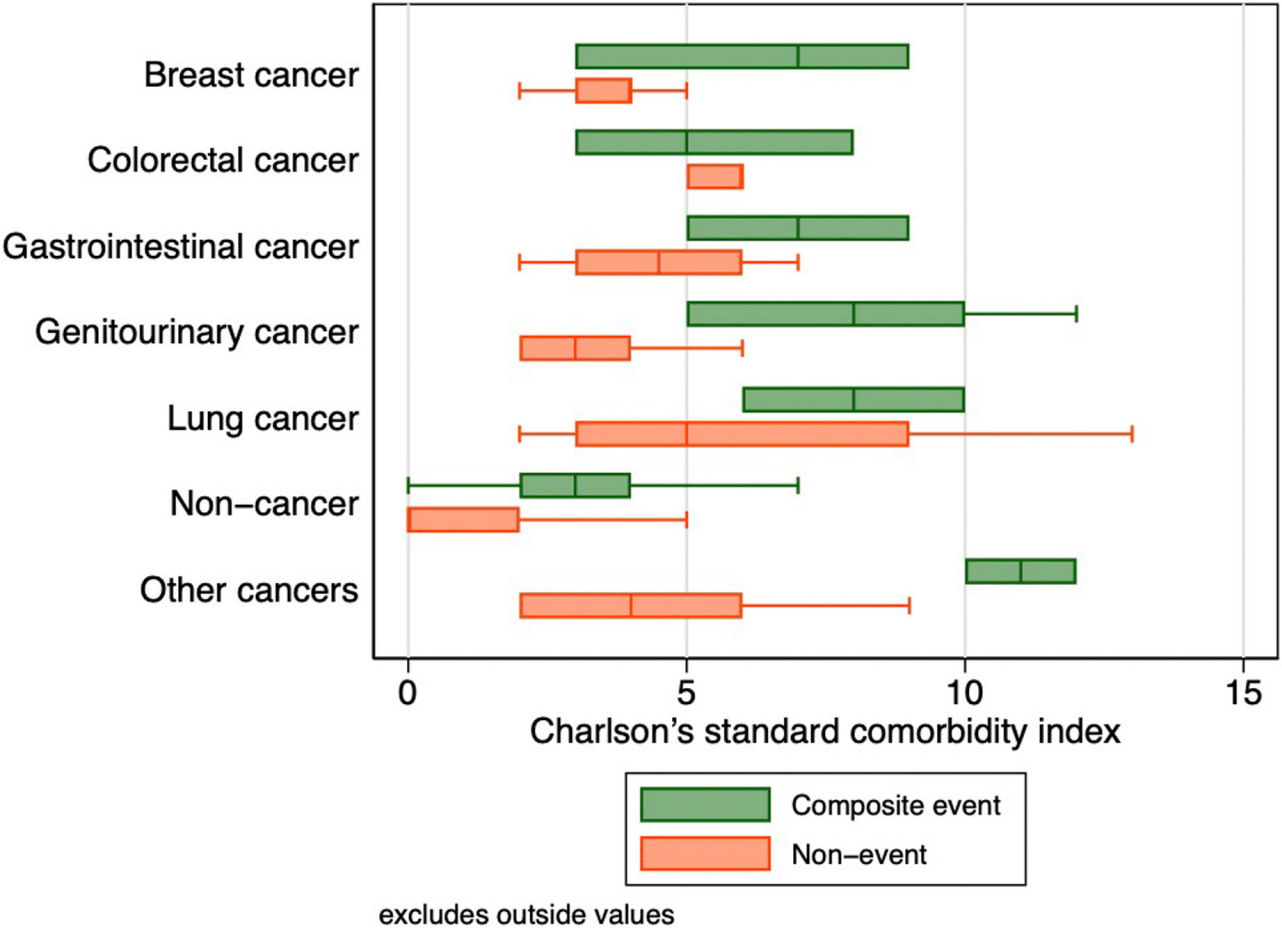
Boxplot of Charlson's comorbidity index for hospitalized COVID‐19 cancer patients who developed the primary outcome.

**FIGURE 2:**
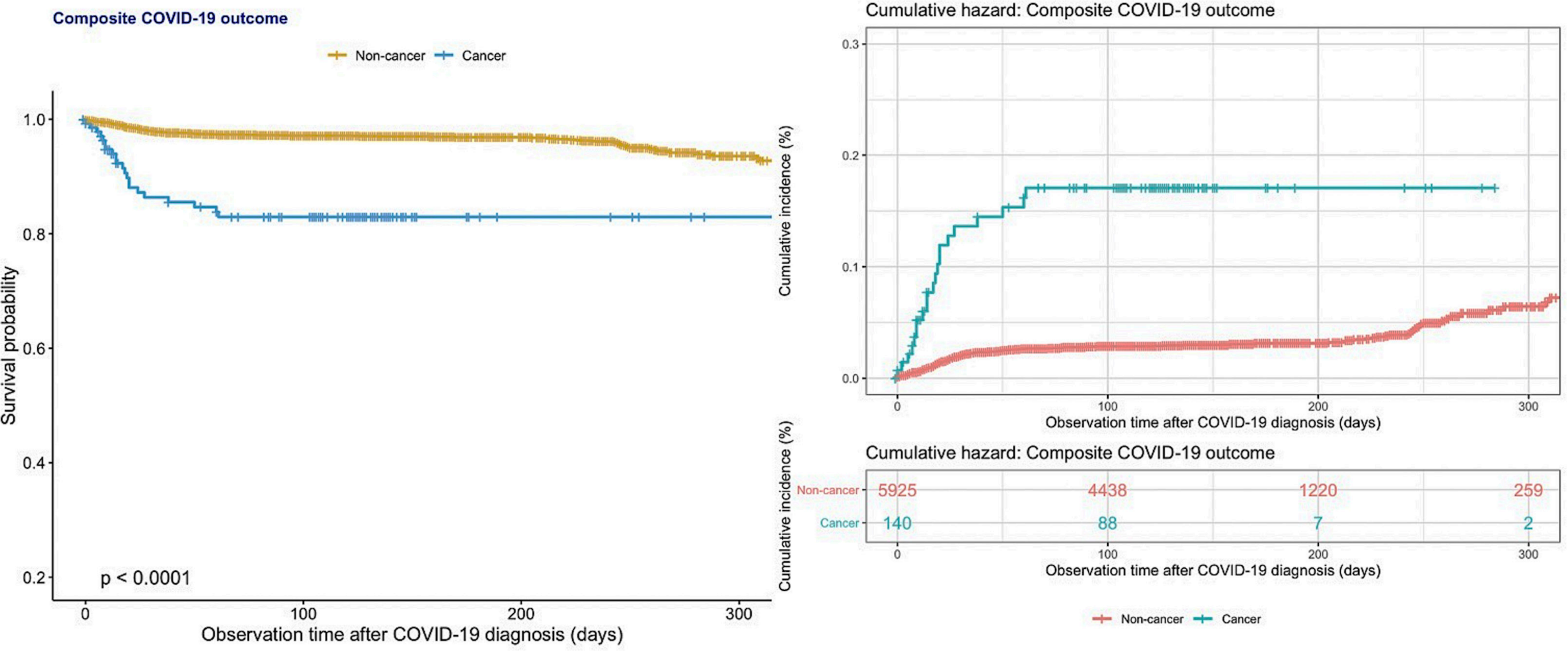
Kaplan–Meier survival curves and cumulative hazards stratified by cancer presentation for severe composite outcome in hospitalized COVID‐19 patients.

A total of 121 patients passed away during the follow‐up period of 12 months, among which a significantly greater proportion were cancer subjects (*p* < 0.05), although there was no difference in the requirement for intubation between the two groups. A comparison between the outcome frequencies between the present study and existing studies has been represented in Table 2. A boxplot showing the mean differences in the Charlson's comorbidity index, which predicts a 10‐year survival rate for patients with a multitude of comorbidities, for the various cancer patients who developed the composite outcome is shown in Figure 1, in turn showcasing the significantly higher comparative comorbidity score of those who developed the composite outcome relative to those who remained outcome free. The results of univariate Cox proportional hazards analysis assessing the relationship between several clinical and laboratory parameters with the composite outcome are shown in Table 3, with Kaplan–Meier survival analysis as shown in Figure 2. Univariate Cox regression revealed that patients with any malignancy at baseline were significantly more likely to experience the composite outcome relative to cancer‐free subjects (HR: 6.32; 95% CI: 4.06–9.85; *p* < 0.001). This relationship held even after subgroup stratification into different types of cancers.

**TABLE 2:** Latest data on mortality rate and ICU admission rates in COVID‐19 cancer and non‐cancer patients. Adapted from^7^

Author	Year	Place	Population	ICU admission	Mortality rate
Guan et al.^27^	2019	China	Non‐cancer (1089)	4.8%	1.4%
			Cancer (10)	30%	0
Yang et al.^28^	2020	China	Non‐cancer (50)	31.7%	15%
			Cancer (2)	0	0
Wang et al.^29^	2020	China	Non‐cancer (128)	25%	—
			Cancer (10)	40%	
Lei et al.^30^	2020	China	Non‐cancer (25)	40%	12%
			Cancer (9)	55.5%	44.4%
Lee et al.^31^	2020	UK	Cancer (1044)	—	28.2%
Mehta et al.^24^	2020	USA	Cancer (218)	—	28%
Shahidsales et al^7^	2020	Iran	Non‐cancer (93)	15.1%	17.2%
			Cancer (92)	24.7%	41.3%
Erdal et al.^32^	2021	Turkey	Non‐cancer (4412)	—	1.51%
			Cancer (77)		23.9%
de Melo et al.^33^	2021	Brazil	Cancer (181)	—	33.1%

**TABLE 3:** Univariate Cox regression analysis for significant risk factors of severe COVID‐19 composite outcome

Characteristics	HR [95% CI]	*p* value
Demographics		
Male gender	1.79 [1.34, 2.38]	0.0001
Baseline age, years	1.07 [1.06, 1.08]	<0.0001
Past comorbidities		
Charlson's standard comorbidity index	1.13 [1.12, 1.15]	<0.0001
Number of comorbidities	1.27 [1.22, 1.31]	<0.0001
Diabetes mellitus	6.14 [4.04, 9.33]	<0.0001
Systemic embolism	—	—
Hypertension	6.76 [5.14, 8.89]	<0.0001
Heart failure	4.68 [1.74, 12.62]	0.0023
Atrial fibrillation	5.81 [3.37, 10.00]	<0.0001
Chronic renal failure	—	—
Liver diseases	2.16 [0.54, 8.72]	0.2780
Ventricular tachycardia/fibrillation	14.27 [7.30, 27.88]	<0.0001
Dementia and Alzheimer	6.37 [2.36, 17.19]	0.0003
AMI	9.16 [5.57, 15.06]	<0.0001
COPD	0.88 [0.22, 3.54]	0.8570
IHD	6.67 [4.59, 9.68]	<0.0001
PVD	6.73 [2.50, 18.13]	0.0002
Stroke/TIA	8.85 [5.86, 13.36]	<0.0001
Gastrointestinal bleeding	7.59 [4.87, 11.81]	<0.0001
Obesity	1.71 [0.24, 12.18]	0.5940
Baseline cancers		
Any cancer	6.32 [4.06, 9.85]	<0.0001
Lung cancer	9.99 [3.19, 31.24]	0.0001
Gastrointestinal cancer	7.43 [2.37, 23.27]	0.0006
Breast cancer	3.35 [1.07, 10.49]	0.0376
Genitourinary cancer	14.60 [4.66, 45.73]	<0.0001
Colorectal cancer	9.89 [3.16, 30.97]	0.0001
Other cancers	5.68 [3.09, 10.45]	<0.0001
Medications		
ACEI	6.93 [4.94, 9.74]	<0.0001
ARB	3.31 [2.12, 5.16]	<0.0001
Calcium channel blockers	6.23 [4.72, 8.22]	<0.0001
Beta blockers	6.45 [4.67, 8.91]	<0.0001
Diuretics for hypertension	2.37 [1.05, 5.35]	0.0373
Diuretics for heart failure	18.91 [14.08, 25.39]	<0.0001
Nitrates	4.97 [2.98, 8.29]	<0.0001
Antihypertensive drugs	5.65 [3.62, 8.80]	<0.0001
Antidiabetic drugs	8.44 [6.29, 11.34]	<0.0001
Lipid‐lowering drugs	6.28 [4.73, 8.35]	<0.0001
Steroid	4.00 [2.97, 5.38]	<0.0001
Lopinavir/ritonavir	1.34 [0.96, 1.87]	0.0906.
Ribavirin	0.97 [0.65, 1.47]	0.8970
Interferon beta‐1B	2.34 [1.73, 3.16]	<0.0001
Proton pump inhibitors	17.04 [12.69, 22.90]	<0.0001
Famotidine	4.28 [3.24, 5.65]	<0.0001
Anticoagulants	26.96 [20.14, 36.08]	<0.0001
Antiplatelets	8.96 [6.75, 11.91]	<0.0001

Abbreviations: ACEI, angiotensinogen‐converting enzyme inhibitor; AMI, acute myocardial infarction; APTT, activated partial thromboplastin time; ARB, angiotensin receptor blocker; COPD, chronic obstructive pulmonary disease; IHD, ischemic heart disease; PVD, peripheral vascular disease; TIA, transient ischemic attack.

As seen in Table 4, after multivariate adjustment for significant demographics, COVID‐19 cancer patients presenting with either any type of cancer, lung cancer, genitourinary cancer, colorectal cancer, or other cancers presented with a higher risk of the composite outcome. Model 2, which additionally adjusted for Charlson's comorbidity index, number of comorbidities and past comorbidities, similarly showed significant associations for patients presenting with any type of cancer, lung cancer, genitourinary cancer, colorectal cancer, or other cancers. Model 3 served as the final stepwise adjustment that included all the aforementioned variables, with the addition of medication treatments. In this model, COVID‐19 patients with any type of cancer presented with an almost four‐fold increase in risk of the composite outcome compared to COVID‐19 subjects without malignancy (HR: 3.77; 95% CI: 1.63–8.72; *p* < 0.002). This association remained significant when assessing those with colorectal (HR: 5.07; 95% CI: 1.50–17.17; *p* < 0.009) and gastrointestinal malignancies (HR: 3.79; 95% CI: 1.12–12.88; *p* < 0.03) relative to their respective cancer‐free COVID‐19 counterparts.

**TABLE 4:** Multivariate Cox adjustments for severe COVID‐19 composite outcome in hospitalized patients

Model	Type of cancer	Adjusted HR [95% CI]	*p* value
Model 1	Any cancer	3.07 [1.97, 4.81]	<0.0001
	Lung cancer	7.19 [2.30, 22.52]	0.0007
	Gastrointestinal cancer	3.13 [1.00, 9.82]	0.0502
	Breast cancer	2.73 [0.86, 8.69]	0.0884
	Genitourinary cancer	9.73 [3.10, 30.54]	0.0001
	Colorectal cancer	5.94 [1.90, 18.63]	0.0022
	Other cancers	2.28 [1.23, 4.21]	0.0085
Model 2	Cancer	7.58 [3.43, 16.75]	<0.0001
	Lung cancer	4.99 [1.32, 18.90]	0.0181
	Gastrointestinal cancer	1.10 [0.27, 4.47]	0.8902
	Breast cancer	2.77 [0.75, 10.23]	0.1268
	Genitourinary cancer	6.77 [1.90, 24.05]	0.0031
	Colorectal cancer	5.38 [1.52, 19.05]	0.0092
	Other cancers	4.07 [1.72, 9.62]	0.0014
Model 3	Cancer	3.77 [1.63, 8.72]	0.0019
	Lung cancer	3.19 [0.88, 11.54]	0.0766
	Gastrointestinal cancer	3.79 [1.12, 12.88]	0.0325
	Breast cancer	2.73 [0.76, 9.79]	0.1222
	Genitourinary cancer	2.63 [0.67, 10.31]	0.1659
	Colorectal cancer	5.07 [1.5, 17.17]	0.0091
	Other cancers	1.34 [0.58, 3.11]	0.4897

*Note*: Model 1: Adjusted for significant demographics.

Model 2: Adjusted for significant demographics, Charlson's comorbidity index, number of comorbidities and past comorbidities.

Model 3: Adjusted for significant demographics, Charlson's comorbidity index, number of comorbidities, past comorbidities and medication treatments.

Abbreviations: ACEI, angiotensinogen‐converting enzyme inhibitor; AMI, acute myocardial infarction; APTT, activated partial thromboplastin time; ARB, angiotensin receptor blocker; COPD, chronic obstructive pulmonary disease; IHD, ischemic heart disease; PVD, peripheral vascular disease; TIA, transient ischemic attack.

## 4 DISCUSSION

The main finding of this study is that COVID‐19 cancer patients have a higher risk of requiring intubation, ICU admission and/or mortality in comparison to those without malignancy. To our knowledge, this is among the first studies to assess outcomes in COVID‐19 cancer in Hong Kong.

The COVID‐19 pandemic caused by SARS‐CoV‐2 has caused significant morbidity and mortality across the globe, inciting many clinical and laboratory investigations concentrated on prevention and management. As it pertains to the former, several patient subgroups have been identified that require attention due to their enhanced susceptibility to infection. In the context of malignancy, an abundance of evidence suggests a weakened immune response as direct sequelae of cancer or secondary to chemotherapy as a fundamental explanation for the observed vulnerability among this patient group.^5^, ^6^ In a nationwide analysis conducted in China, the COVID‐19 cohort presented with a greater proportion of cancer patients relative to the general Chinese population per 100,000 people,^8^ among which lung cancer was the most common type. The findings of another retrospective cohort study lend further credence to this notion by demonstrating that those with stage IV cancer had the highest infection rate compared to the lower stages.^14^ Regarding clinical presentation, Although these subjects tend to present with similar symptoms as their respective cancer‐free counterparts, specifically fever, fatigue, cough and dyspnea,^14^, ^15^ patients with malignancy more frequently progress to severe complications,^5^ ranging from liver injury^16^ and acute respiratory distress syndrome^17^ to various cardiovascular conditions, chiefly acute myocardial infarction, arrhythmias, stroke and embolism.^7^, ^18^, ^19^, ^20^ Thus, this has important clinical implications: cancer patients who suffer from COVID‐19 would require more extensive monitoring of their clinical statuses such as white blood cell counts, inflammatory markers, functions of cardiovascular, respiratory systems, and liver and renal function as well as adjusting their treatment regime as appropriate.

Numerous studies have examined the prognostic outcomes of cancer patients with COVID‐19. As with our investigation, the aforementioned nationwide study in China showcased that those diagnosed with malignancy were predisposed to a similar composite endpoint of ICU admission, requirement for intensive ventilation and/or death, although the number of cancer subjects analyzed in this study was much smaller (*n* = 18).^8^ Moreover, a study conducted in Wuhan, China that recruited only those with haematological malignancies also displayed a more severe symptomatic presentation, disease course and case fatality rate within the patient group compared to cancer‐free subjects.^21^ Our data are also supported by several other investigations performed in other countries with non‐Chinese cohorts that have reported findings of a bleaker prognosis for infected cancer patients.^22^, ^23^, ^24^, ^25^, ^26^ An overview of such results is also summarized in Table 4 which provides the comparison of mortality and ICU admission rates in COVID‐19 cancer and non‐cancer patients, which the former had demonstrated more adverse outcomes. In addition, these results have been appropriately summated in a systematic review and meta‐analysis that compiled the different observed outcomes experienced by infected subjects in various localities to likewise confirm their findings of more severe long‐term outcomes for those with malignancy using a combined sample size of 1018 cancer patients.

### 4.1 Limitations

This study has several limitations that should be noted in terms of its type of study, selection bias and sampling bias and confounding. First, its retrospective nature prevents the analysis of clinical outcomes during real‐time follow‐up. Second, it is limited by significant selection bias. To illustrate, the cohort consisted of patients recruited from a single locality, Hong Kong, therefore is unable to account for any geographical heterogeneity that may persist among COVID‐19 cancer subjects. The study population also only included patients who had presented to the public healthcare system in Hong Kong leading to admission bias. Furthermore, the included patients also had several other comorbidities that likely had an influence on the composite outcome risk. Third, the sample size is too small, which may have contributed to statistical imprecision. This could in turn perhaps explain the lack of statistical significance between either lung or genitourinary cancer and the risk of composite outcome. Moreover, patients were also not further stratified based on their cancer staging and the anticancer treatment that the patients were receiving at the time of the COVID‐19 diagnosis, making it difficult to assess the degree of immunosuppression of the patients which would have contributed to the composite outcome. Finally, another important limitation is the use of a composite outcome, which is the strategy that has been adopted by much of the existing literature. With the assessment of a combined outcome, it is difficult to ascertain how much the individual components that constitute the endpoint have contributed to the outcome. Cancer patients already have a higher risk of mortality than non‐cancer subjects. As such, the observed differences in the endpoint between the two groups may simply reflect the preexisting, inherent differences in mortality risk due to the cancer status, as opposed to COVID‐19 infection status.

## 5 CONCLUSION

COVID‐19 cancer patients are associated with a significantly higher risk of intubation, ICU admission and mortality. Given their inherent susceptibility to infection, coupled with the evident worse prognosis, these subjects should receive dedicated clinical attention to improve their long‐term outcomes.

### AUTHOR CONTRIBUTION

JZ, IL: data analysis, data interpretation, statistical analysis, manuscript drafting and critical revision of the manuscript. OC, KSKL, TT, LL, ZL, AKCW, CC, ICKW: project planning, data acquisition, data interpretation and critical revision of the manuscript. QZ, GT, BMYC: study conception, study supervision, project planning, data interpretation, statistical analysis, manuscript drafting and critical revision of the manuscript.

## FUNDING INFORMATION

None.

## CONFLICT OF INTEREST

The authors have nothing to disclose.

## Supporting information


Table S1‐S2

## Data Availability

The data that support the findings of this study are available on request from the corresponding author. The data are not publicly available due to privacy or ethical restrictions.
