# Supplementary material for: Clinical characteristics, risk factors and outcomes of cancer patients with COVID‐19: A population‐based study
Source: Cancer Med. 2022 May 31;12(1):287–96. doi: 10.1002/cam4.4888 (PMC9348317; doi:10.1002/cam4.4888)
Supplement: Supplementary file 1 — Table S1‐S2 [file CAM4-12-287-s001.docx]

**Supplementary Table 1. Baseline and clinical characteristics of hospitalized COVID-19 patients stratified by lung cancer, gastrointestinal cancer, and breast cancer**

AMI: acute myocardial infarction; COPD: chronic obstructive pulmonary disease; IHD: ischemic heart disease; PVD: peripheral vascular disease; TIA: transient ischemic attack; ACEI: angiotensinogen converting enzyme inhibitor; ARB: angiotensin receptor blocker; APTT: activated partial thromboplastin time.

| **Characteristics** | **No cancer (N=5947)**  **Median (IQR) or Count (%)** | **Lung cancer (N=9)**  **Median (IQR) or Count (%)** | **P value** | **Gastrointestinal cancer (N=14)**  **Median (IQR) or Count (%)** | **P value** | **Breast cancer (N=27) Median (IQR) or Count (%)** | **P value** |
| --- | --- | --- | --- | --- | --- | --- | --- |
| ***Demographics*** |  |  |  |  |  |  |  |
| Male gender | 2966(49.87) | 6(66.66) | 0.7772 | 9(64.28) | 0.7038 | 0(0.00) | 0.0005 |
| Baseline age, years | 45.2(27.6-62.33) | 63.78(59.89-71.03) | 0.0065 | 67.99(62.95-74.38) | <0.0001 | 60.41(56.46-67.44) | <0.0001 |
| ***Past comorbidities*** |  |  |  |  |  |  |  |
| Charlson score | 0.0(0.0-2.0) | 6.0(5.0-10.0) | <0.0001 | 4.5(4.0-6.5) | <0.0001 | 4.0(3.0-4.0) | <0.0001 |
| Number of comorbidities | 0.0(0.0-0.0) | 1.0(1.0-2.0) | <0.0001 | 2.0(1.0-2.0) | <0.0001 | 1.0(1.0-2.0) | <0.0001 |
| Diabetes mellitus | 132(2.21) | 2(22.22) | 0.0119 | 0(0.00) | 0.7494 | 2(7.40) | 0.2995 |
| Systemic embolism | 16(0.26) | 0(0.00) | 0.0026 | 0(0.00) | 0.0192 | 0(0.00) | 0.1199 |
| Hypertension | 845(14.20) | 0(0.00) | 0.5122 | 7(50.00) | 0.0132 | 9(33.33) | 0.0535 |
| Heart failure | 31(0.52) | 0(0.00) | 0.0362 | 0(0.00) | 0.1133 | 0(0.00) | 0.3372 |
| Atrial fibrillation | 84(1.41) | 1(11.11) | 0.3445 | 1(7.14) | 0.5379 | 1(3.70) | 0.8779 |
| Chronic renal failure | 15(0.25) | 0(0.00) | 0.0019 | 0(0.00) | 0.0153 | 0(0.00) | 0.1054 |
| Liver diseases | 29(0.48) | 0(0.00) | 0.0362 | 0(0.00) | 0.1133 | 0(0.00) | 0.3372 |
| Ventricular tachycardia/fibrillation | 28(0.47) | 0(0.00) | 0.0327 | 1(7.14) | 0.1226 | 0(0.00) | 0.3237 |
| Dementia and Alzheimer | 22(0.36) | 0(0.00) | 0.0155 | 0(0.00) | 0.0634 | 1(3.70) | 0.2525 |
| AMI | 68(1.14) | 2(22.22) | 0.0002 | 1(7.14) | 0.4477 | 1(3.70) | 0.7758 |
| COPD | 75(1.26) | 0(0.00) | 0.2631 | 0(0.00) | 0.4573 | 1(3.70) | 0.8256 |
| IHD | 180(3.02) | 3(33.33) | 0.0004 | 2(14.28) | 0.1494 | 2(7.40) | 0.5236 |
| PVD | 25(0.42) | 0(0.00) | 0.0206 | 0(0.00) | 0.077 | 0(0.00) | 0.2679 |
| Stroke/TIA | 112(1.88) | 1(11.11) | 0.4847 | 1(7.14) | 0.6976 | 3(11.11) | 0.0112 |
| Gastrointestinal bleeding | 103(1.73) | 1(11.11) | 0.4604 | 3(21.42) | <0.0001 | 1(3.70) | 0.9787 |
| Obesity | 23(0.38) | 0(0.00) | 0.0132 | 0(0.00) | 0.0569 | 0(0.00) | 0.2243 |
| ***Medications*** |  |  |  |  |  |  |  |
| ACEI | 198(3.32) | 0(0.00) | 0.6949 | 1(7.14) | 0.984 | 1(3.70) | 0.6628 |
| ARB | 179(3.00) | 0(0.00) | 0.6575 | 2(14.28) | 0.1377 | 3(11.11) | 0.0889 |
| Calcium channel blockers | 562(9.45) | 1(11.11) | 0.6663 | 5(35.71) | 0.0230 | 5(18.51) | 0.3009 |
| Beta blockers | 245(4.11) | 2(22.22) | 0.1124 | 2(14.28) | 0.2904 | 4(14.81) | 0.0443 |
| Diuretics for hypertension | 61(1.02) | 1(11.11) | 0.2167 | 0(0.00) | 0.35 | 0(0.00) | 0.6769 |
| Diuretics for heart failure | 134(2.25) | 2(22.22) | 0.0132 | 1(7.14) | 0.7993 | 2(7.40) | 0.3123 |
| Nitrates | 86(1.44) | 2(22.22) | 0.0007 | 2(14.28) | 0.0081 | 0(0.00) | 0.8636 |
| Antihypertensive drugs | 101(1.69) | 0(0.00) | 0.3858 | 1(7.14) | 0.6423 | 0(0.00) | 0.9705 |
| Antidiabetic drugs | 274(4.60) | 2(22.22) | 0.1514 | 2(14.28) | 0.3599 | 3(11.11) | 0.3263 |
| Lipid-lowering drugs | 465(7.81) | 2(22.22) | 0.4239 | 2(14.28) | 0.7559 | 3(11.11) | 0.836 |
| Steroid | 545(9.16) | 2(22.22) | 0.5401 | 3(21.42) | 0.3573 | 3(11.11) | 0.9752 |
| Lopinavir/ritonavir | 812(13.65) | 0(0.00) | 0.553 | 1(7.14) | 0.8139 | 6(22.22) | 0.4048 |
| Ribavirin | 624(10.49) | 1(11.11) | 0.6257 | 2(14.28) | 0.9837 | 6(22.22) | 0.1612 |
| Interferon beta-1B | 847(14.24) | 2(22.22) | 0.9156 | 4(28.57) | 0.379 | 8(29.62) | 0.1134 |
| Proton pump inhibitors | 768(12.91) | 3(33.33) | 0.3374 | 6(42.85) | 0.0306 | 6(22.22) | 0.3944 |
| Famotidine | 811(13.63) | 2(22.22) | 0.8862 | 0(0.00) | 0.3225 | 13(48.14) | 0.0002 |
| Anticoagulants | 468(7.86) | 4(44.44) | 0.0083 | 3(21.42) | 0.2658 | 3(11.11) | 0.8749 |
| Antiplatelets | 381(6.40) | 1(11.11) | 0.8769 | 2(14.28) | 0.595 | 5(18.51) | 0.0633 |
| ***Complete blood counts*** |  |  |  |  |  |  |  |
| Mean corpuscular volume, fL | 87.2(83.5-90.4) | 86.6(85.3-88.15) | 0.6903 | 88.8(85.6-92.45) | 0.3481 | 84.35(81.05-89.15) | 0.1328 |
| Basophil, x10^9/L | 0.01(0.0-0.02) | 0.02(0.0-0.03) | 0.615 | 0.01(0.0-0.02) | 0.9088 | 0.01(0.0-0.01) | 0.3175 |
| Eosinophil, x10^9/L | 0.03(0.0-0.1) | 0.02(0.01-0.11) | 0.6053 | 0.02(0.0-0.07) | 0.616 | 0.01(0.0-0.09) | 0.2791 |
| Lymphocyte, x10^9/L | 1.33(0.97-1.8) | 0.7(0.61-0.78) | 0.0133 | 1.16(0.7-1.82) | 0.3781 | 1.07(0.84-1.44) | 0.0255 |
| Blast, x10^9/L | 0.0(0.0-0.0) | 0.1(0.1-0.1) | 0.0029 | 0.0(0.0-0.0) | 0.7517 | 0.0(0.0-0.0) | 0.7517 |
| Metamyelocyte, x10^9/L | 0.1(0.07-0.17) | - | - | 0.27(0.27-0.27) | 0.0861 | - | - |
| Monocyte, x10^9/L | 0.5(0.37-0.63) | 0.52(0.42-0.7) | 0.4247 | 0.4(0.34-0.72) | 0.6877 | 0.5(0.38-0.6) | 0.9433 |
| Neutrophil, x10^9/L | 3.2(2.37-4.34) | 4.8(3.39-7.18) | 0.0699 | 3.66(2.86-5.08) | 0.2518 | 2.79(2.28-4.31) | 0.4962 |
| White cell count, x10^9/L | 5.34(4.24-6.71) | 6.4(5.0-9.11) | 0.2454 | 5.76(4.93-6.59) | 0.4898 | 4.62(3.92-6.03) | 0.1275 |
| Mean cell haemoglobin, pg | 29.9(28.5-31.18) | 28.8(27.95-31.05) | 0.6442 | 31.7(30.35-32.8) | 0.0069 | 31.45(28.9-34.5) | 0.0514 |
| Myelocyte, x10^9/L | 0.22(0.09-0.37) | - | - | - | - | 0.15(0.15-0.15) | 0.8364 |
| Platelet, x10^9/L | 215.0(174.0-267.85) | 258.5(190.5-302.5) | 0.5416 | 185.0(152.0-217.5) | 0.1108 | 191.0(164.5-226.5) | 0.1093 |
| Reticulocyte, x10^9/L | 42.6(30.2-71.5) | - | - | - | - | - | - |
| Red blood count, x10^12/L | 4.65(4.32-5.05) | 4.57(4.04-5.2) | 0.8098 | 4.56(3.78-4.71) | 0.0972 | 4.36(4.09-4.72) | 0.0117 |
| Hematocrit, L/L | 0.4(0.37-0.43) | - | - | - | - | 0.37(0.36-0.39) | 0.0764 |

**Supplementary Table 2. Baseline and clinical characteristics of hospitalized COVID-19 patients stratified by genitourinary cancer, colorectal cancer, and other cancers.**

AMI: acute myocardial infarction; COPD: chronic obstructive pulmonary disease; IHD: ischemic heart disease; PVD: peripheral vascular disease; TIA: transient ischemic attack; ACEI: angiotensinogen converting enzyme inhibitor; ARB: angiotensin receptor blocker; APTT: activated partial thromboplastin time.

| **Characteristics** | **No Cancer (N=5947) Median (IQR) or Count (%)** | **Genitourinary cancer (N=8)**  **Median (IQR) or Count (%)** | **P value** | **Colorectal cancer (N=11)**  **Median (IQR) Count (%)** | **P value** | **Other cancers (N=85) Median (IQR) or Count (%)** | **P value** |
| --- | --- | --- | --- | --- | --- | --- | --- |
| ***Demographics*** |  |  |  |  |  |  |  |
| Male gender | 2966(49.87) | 4(50.00) | 0.7651 | 7(63.63) | 0.7949 | 46(54.11) | 0.7125 |
| Baseline age, years | 45.22(27.62-62.33) | 59.89(49.28-80.01) | 0.0681 | 64.46(61.52-69.3) | 0.0007 | 65.42(39.38-82.59) | <0.0001 |
| ***Past comorbidities*** |  |  |  |  |  |  |  |
| Charlson score | 0.0(0.0-2.0) | 3.5(2.5-9.0) | <0.0001 | 4.0(4.0-6.5) | <0.0001 | 4.0(2.0-6.0) | <0.0001 |
| Number of comorbidities | 0.0(0.0-0.0) | 2.0(1.0-2.5) | <0.0001 | 2.0(1.0-2.0) | <0.0001 | 1.0(1.0-2.0) | <0.0001 |
| Diabetes mellitus | 132(2.21) | 2(25.00) | 0.0072 | 0(0.00) | 0.6178 | 6(7.05) | 0.0161 |
| Systemic embolism | 16(0.26) | 0(0.00) | 0.0013 | 0(0.00) | 0.0072 | 1(1.17) | 0.5912 |
| Hypertension | 845(14.20) | 3(37.50) | 0.3282 | 4(36.36) | 0.2254 | 35(41.17) | <0.0001 |
| Heart failure | 31(0.52) | 0(0.00) | 0.0246 | 0(0.00) | 0.0641 | 1(1.17) | 0.9395 |
| Atrial fibrillation | 84(1.41) | 1(12.50) | 0.2998 | 0(0.00) | 0.3871 | 2(2.35) | 0.8231 |
| Chronic renal failure | 15(0.25) | 0(0.00) | 0.0009 | 0(0.00) | 0.0054 | 1(1.17) | 0.5597 |
| Liver diseases | 29(0.48) | 0(0.00) | 0.0246 | 0(0.00) | 0.0641 | 3(3.52) | 0.0024 |
| Ventricular tachycardia/fibrillation | 28(0.47) | 0(0.00) | 0.0220 | 1(9.09) | 0.0738 | 2(2.35) | 0.1071 |
| Dementia and Alzheimer | 22(0.36) | 0(0.00) | 0.0096 | 0(0.00) | 0.0314 | 2(2.35) | 0.0528 |
| AMI | 68(1.14) | 1(12.50) | 0.23 | 1(9.09) | 0.345 | 2(2.35) | 0.6517 |
| COPD | 75(1.26) | 0(0.00) | 0.2196 | 0(0.00) | 0.3458 | 5(5.88) | 0.0020 |
| IHD | 180(3.02) | 1(12.50) | 0.6738 | 1(9.09) | 0.8344 | 9(10.58) | 0.0009 |
| PVD | 25(0.42) | 0(0.00) | 0.0131 | 0(0.00) | 0.0399 | 2(2.35) | 0.0691 |
| Stroke/TIA | 112(1.88) | 0(0.00) | 0.3784 | 0(0.00) | 0.5328 | 4(4.70) | 0.1726 |
| Gastrointestinal bleeding | 103(1.73) | 2(25.00) | 0.0020 | 2(18.18) | 0.0096 | 6(7.05) | 0.0028 |
| Obesity | 23(0.38) | 0(0.00) | 0.0080 | 0(0.00) | 0.0275 | 1(1.17) | 0.778 |
| ***Medications*** |  |  |  |  |  |  |  |
| ACEI | 198(3.32) | 0(0.00) | 0.6307 | 0(0.00) | 0.8069 | 4(4.70) | 0.7109 |
| ARB | 179(3.00) | 2(25.00) | 0.0270 | 2(18.18) | 0.0729 | 4(4.70) | 0.615 |
| Calcium channel blockers | 562(9.45) | 2(25.00) | 0.4955 | 3(27.27) | 0.2368 | 18(21.17) | 0.0035 |
| Beta blockers | 245(4.11) | 1(12.50) | 0.8291 | 1(9.09) | 0.9943 | 8(9.41) | 0.0543 |
| Diuretics for hypertension | 61(1.02) | 0(0.00) | 0.1461 | 0(0.00) | 0.2514 | 2(2.35) | 0.5265 |
| Diuretics for heart failure | 134(2.25) | 1(12.50) | 0.5234 | 1(9.09) | 0.6756 | 6(7.05) | 0.0186 |
| Nitrates | 86(1.44) | 0(0.00) | 0.2575 | 1(9.09) | 0.4328 | 0(0.00) | 0.501 |
| Antihypertensive drugs | 101(1.69) | 0(0.00) | 0.3335 | 0(0.00) | 0.4815 | 7(8.23) | <0.0001 |
| Antidiabetic drugs | 274(4.60) | 1(12.50) | 0.89 | 2(18.18) | 0.2309 | 11(12.94) | 0.0028 |
| Lipid-lowering drugs | 465(7.81) | 3(37.50) | 0.0504 | 0(0.00) | 0.7213 | 8(9.41) | 0.7901 |
| Steroid | 545(9.16) | 0(0.00) | 0.82 | 2(18.18) | 0.6932 | 12(14.11) | 0.2311 |
| Lopinavir/ritonavir | 812(13.65) | 1(12.50) | 0.6641 | 1(9.09) | 0.9549 | 8(9.41) | 0.3972 |
| Ribavirin | 624(10.49) | 0(0.00) | 0.7522 | 2(18.18) | 0.8044 | 8(9.41) | 0.8987 |
| Interferon beta-1B | 847(14.24) | 0(0.00) | 0.5892 | 3(27.27) | 0.5509 | 15(17.64) | 0.5592 |
| Proton pump inhibitors | 768(12.91) | 4(50.00) | 0.064 | 4(36.36) | 0.17 | 35(41.17) | <0.0001 |
| Famotidine | 811(13.63) | 2(25.00) | 0.7886 | 0(0.00) | 0.4365 | 20(23.52) | 0.0454 |
| Anticoagulants | 468(7.86) | 3(37.50) | 0.0573 | 3(27.27) | 0.1455 | 19(22.35) | <0.0001 |
| Antiplatelets | 381(6.40) | 0(0.00) | 0.9933 | 1(9.09) | 0.772 | 12(14.11) | 0.0193 |
| ***Complete blood counts*** |  |  |  |  |  |  |  |
| Mean corpuscular volume, fL | 87.2(83.5-90.4) | 88.6(86.45-92.05) | 0.2937 | 87.35(85.6-90.9) | 0.9091 | 88.9(84.9-92.6) | 0.0058 |
| Basophil, x10^9/L | 0.01(0.0-0.02) | 0.0(0.0-0.01) | 0.2984 | 0.01(0.0-0.02) | 0.9598 | 0.01(0.0-0.02) | 0.2312 |
| Eosinophil, x10^9/L | 0.03(0.0-0.1) | 0.0(0.0-0.1) | 0.817 | 0.03(0.0-0.07) | 0.8065 | 0.02(0.0-0.1) | 0.5012 |
| Lymphocyte, x10^9/L | 1.33(0.97-1.8) | 1.2(0.68-1.32) | 0.1146 | 1.4(0.96-1.82) | 0.9719 | 1.1(0.78-1.5) | 0.0002 |
| Blast, x10^9/L | 0.0(0.0-0.0) | - | - | 0.0(0.0-0.0) | 0.7517 | 0.0(0.0-0.0) | 0.5101 |
| Metamyelocyte, x10^9/L | 0.1(0.07-0.17) | - | - | 0.31(0.31-0.31) | 0.184 | 0.02(0.02-0.02); | 0.1253 |
| Monocyte, x10^9/L | 0.5(0.37-0.63) | 0.3(0.25-0.6) | 0.1909 | 0.5(0.35-0.72) | 0.924 | 0.5(0.37-0.71) | 0.4147 |
| Neutrophil, x10^9/L | 3.2(2.37-4.34) | 2.7(2.16-3.55) | 0.4287 | 3.96(3.03-5.08) | 0.2351 | 3.29(2.43-4.51) | 0.4678 |
| White cell count, x10^9/L | 5.34(4.24-6.71) | 4.4(3.31-5.25) | 0.1546 | 5.94(5.52-6.59) | 0.2317 | 5.3(3.99-6.44) | 0.6162 |
| Mean cell haemoglobin, pg | 29.9(28.5-31.18) | 30.7(29.45-31.2) | 0.3692 | 31.6(30.35-32.4) | 0.0272 | 30.0(29.0-31.7) | 0.1946 |
| Myelocyte, x10^9/L | 0.22(0.09-0.37) | 0.03(0.03-0.03) | 0.1166 | - | - | 0.04(0.04-0.08) | 0.0401 |
| Platelet, x10^9/L | 215.0(174.0-267.85) | 227.0(178.5-259.5) | 0.9128 | 192.0(177.5-217.5) | 0.3656 | 203.0(151.0-267.0) | 0.2686 |
| Reticulocyte, x10^9/L | 42.6(30.2-71.5) | - | - | - | - | 71.49(50.39-86.84) | 0.3115 |
| Red blood count, x10^12/L | 4.65(4.32-5.05) | 4.32(4.2-4.6) | 0.0683 | 4.56(4.1-4.71) | 0.2002 | 4.14(3.73-4.52) | <0.0001 |
| Hematocrit, L/L | 0.4(0.37-0.43) | - | - | - | - | 0.35(0.32-0.38) | <0.0001 |
